# Relationship between *TNF-α* −1031T/C gene polymorphism, plasma level of TNF-α, and risk of cachexia in head and neck cancer patients

**DOI:** 10.1007/s00432-018-2679-4

**Published:** 2018-05-25

**Authors:** Tomasz Powrózek, Radosław Mlak, Anna Brzozowska, Marcin Mazurek, Paweł Gołębiowski, Teresa Małecka-Massalska

**Affiliations:** 1 Department of Human Physiology, Medical University of Lublin, Radziwiłłowska 11, 20-080 Lublin, Poland; 2 Department of Oncology, Medical University of Lublin, Lublin, Poland

**Keywords:** Head and neck cancer, Cachexia, Single-nucleotide polymorphism, TNF-α

## Abstract

**Background:**

Malnutrition and cachexia are frequent among head and neck cancer (HNC) patients and these syndromes are associated with both poor quality of life and unfavorable disease prognosis. Unfortunately, there are still no established biomarkers that could predict the development of cachexia. Among potential molecular alterations related to cancer cachexia, there are single-nucleotide polymorphisms (SNPs) within genes encoding pro-inflammatory cytokines such as TNF-α.

**The aim of the study:**

To investigate *TNF-α* −1031T/C SNP as a risk factor of cachexia in 62 HNC patients subjected to radiotherapy. DNA was isolated from whole blood samples and genotyping was conducted using real-time PCR method by means of TaqMan SNP Genotyping Assay. TNF-alpha Human ELISA Kit was used to determine TNF-α concentration in each extracted plasma sample. Moreover, the relationship between genotype variants of *TNF-α* and plasma level of TNF-α was examined. Detailed clinical–demographic and nutritional data were collected from each study participant.

**Results:**

CC genotype carriers were at a significantly higher risk of being qualified as cachectic compared with other genotype carriers (*p* = 0.044; HR = 3.724). Subjects, who carried CC genotype had significantly lower body mass compared to patients with TT and CT genotype (*p* = 0.045). Moreover, CC individuals had the highest TNF-α plasma level (median 10.70 ± 0.72 pg/mL, *p* = 0.006) among the studied cases. We also noted, that CC genotype carriers had significantly higher risk of early death incidence compared to other genotype carriers [overall survival (OS): 28 vs 38 months (HR = 3.630, *p* = 0.013)].

**Conclusion:**

Despite the differences between SGA and NRS scoring, the presence of CC genotype could be a useful objective marker allowing for the prediction of cachexia development in both parenterally nourished and non-parenterally nourished patients. Patients with CC genotype had also the highest risk of early death incidence; therefore, such individuals should be qualified for parenteral nutrition and supportive care at the time of diagnosis to improve further therapy outcomes. Moreover, this is the first study demonstrating the relationship between *TNF-α* −1031T/C polymorphism and plasma level of TNF-α. This is also the first paper investigating the role of *TNF-α* −1031T/C in cancer cachexia.

## Introduction

The majority of head and neck cancers (HNC) represent a heterogeneous group of squamous-cell-type tumors located in the area of upper aerodigestive tract mucosa. HNC is the sixth most common cancer worldwide with approximately 600,000 new cases diagnosed annually resulting in more than 350,000 deaths every year (Siegel et al. 2016; Sanderson and Ironside 2002). The anatomic location of the tumor usually impedes or inhibits proper patient nutrition; hence, problems with proper ingestion are noted in even up to 50% of HNC patients. In such individuals, undernutrition is frequently present at the time of diagnosis and it can be the first observable symptom of the disease. Despite the fact that most HNC patients undergo radical surgery, radiotherapy, chemotherapy or a combination of these methods, they experience side-effects of the applied therapy, which further contribute to the development of malnutrition (44–88% of HNC patients) (O’Neill and Shaha 2011; Alshadwi et al. 2013; Unsal et al. 2006). Moreover, both malnutrition and noted nutritional deficits have a significant negative impact on the patients’ quality of life and contribute to higher cancer mortality rate. The following symptoms are frequently observed in HNC patients: dysphagia, anorexia, fatigue, and muscle weakness secondary to cancer cachexia. Cachexia is a complex multifactorial syndrome strongly associated with severe metabolic abnormalities characterized by skeletal muscle loss and increased lipolysis that cause weight loss of various degrees. Cachexia is usually accompanied by anorexia and related to overproduction of pro-inflammatory cytokines, such as interleukins and tumor necrosis factor (TNF). The above-mentioned fact emphasizes crucial role of the inflammatory response in the pathogenesis of cachexia (Alshadwi et al. 2013; Gorenc et al. 2015; Ravasco et al. 2003; Tisdale 2009). Currently, the molecular background of cachexia is still unknown, and therefore, it is difficult to identify patients who are likely to be cachectic.

Among potential mechanisms involved in the development of cachexia, the primary initial process is probably the systemic inflammatory response followed by increased production of pro-inflammatory cytokines, such as TNF-α. Multiple biological activities of TNF-α were found in numerous physiological states, including the regulation of cell differentiation, proliferation, apoptosis and metabolism (Locksley et al. 2001; Bazzoni and Beutler 1996). On the other hand, TNF-α was also recognized as a significant regulator of both inflammatory and autoimmune diseases and, moreover, it was implicated in inflammatory-associated tumors and the development of cachexia (Gorenc et al. 2015; Beutler 1999). Up until now, several functional single-nucleotide polymorphisms (SNPs) within *TNF-α* gene have been identified and described as cancer related genetic alterations. The most important ones seem to be SNPs located within the promoter of *TNF-α* because of their ability to regulate gene expression and, consequently, the expression of the TNF-α protein. Among frequently investigated SNPs, the -308 G/A and -238 G/A are potentially involved in tumor aggressiveness, prognosis and risk of malnutrition (Hajeer and Hutchinson 2000; Liu et al. 2005; Hu et al. 2014). There are only few data concerning the role of *TNF-α* −1031T/C SNP (rs1799964) in the regulation of systemic inflammatory response; however, the latest studies have demonstrated the role of this SNP as cachexia related genetic alteration (Johns et al. 2017; Nourian et al. 2017). The significant role of the systemic inflammatory response mediated by TNF-α in the etiopathology of cachexia encourages investigating SNPs of *TNF-α* as cachexia related risk factors. The aim of the study was the investigation of *TNF-α* −1031T/C as a cachexia risk factor as well as the assessment of the correlation between *TNF-α* −1031T/C and plasma TNF-α concentration in HNC patients.

## Materials and methods

### Study group

62 HNC therapy naive patients scheduled to radical radiotherapy (RTH) (51 male and 11 female; median age: 63 ± 8.2 years) were enrolled in the study group. All patients were diagnosed and treated between 2014 and 2015 at the Department of Oncology, Medical University of Lublin. In the studied individuals, alcohol consumption level was evaluated using International Statistical Classification of Diseases and Related Health Problems (ICD). The 7th edition of TNM scale was used to assess the disease stage. The patients’ performance status was assessed according to the Eastern Cooperative Oncology Group–World Health Organization (ECOG–WHO) scale (from 0 to 5; with 0 denoting perfect health and 5 deaths). ECOG–WHO scale assesses how a patient’s disease is progressing and how the disease affects the daily living capabilities of the patient. The detailed clinical and demographic characteristics of the patients are summarized in Table 1. The ONCOR (Siemens) linear accelerator was applied for the radical radiotherapy with the administration of the total doses of 66–70 Gy (daily dose of 2 Gy using IMRT technique).

**Table 1:** Clinical–demographic characteristics and nutritional status of studied patients

Factor	Study group (*n* = 62)
Gender	
Male	51 (82.2%)
Female	11 (17.8%)
Age, median (range)	63 (42–87)
≥ 63	32 (48.4%
< 63	30 (51.6%)
Histopathological diagnosis	
Squamous-cell carcinoma	57 (91.9%)
Other	5 (8.1%)
Tumor location	
Upper throat	17 (27.4%)
Lower throat	45 (72.6%)
Larynx	34 (54.8%)
Others	28 (45.2%)
T stage	
T1	2 (3.2%)
T2	9 (14.5%)
T3	15 (24.2%)
T4	36 (58.1%)
N stage	
Nx	2 (3.2%)
N0	18 (29%)
N1	6 (9.7%)
N2	32(51.6%)
N3	4 (6.5%)
M stage	
Mx	3(75%)
M1	1 (25%)
Disease stage	
I	2 (3.2%)
III	12 (19.4%)
IVA	40 (64.5%)
IVB	3 (4.8%)
IVC	5 (8.1%)
Performance status (PS)	
≤ 1	55 (88.7%)
> 1	7 (11.3%)
Type of treatment	
Surgery + RTH	28 (45.2%)
Surgery + chemoradiation	17 (27.4%)
RTH alone	7 (11.3%)
Induction CHTH + RTH	3 (4.8%)
Concurrent chemoradiation	7 (11.3%)
Alcohol consumption	
Yes	28 (45.2%)
No	34 (54.8%)
Smoking status	
Smoker	52 (83.9%)
Non-smoker	10 (16.1%)
Current smoker	45 (86.5%)
Former smoker	7 (13.5%)
Nutritional status	
Parenteral nutrition	
Yes	13 (21%)
No	49 (79%)
Weight (kg)	
Mean ± SD	65.63 ± 11.93
BMI	
Mean ± SD	23.13 ± 4.44
> 18.5	52 (83.9%)
< 18.5	10 (16.1%)
SGA	
A	9 (14.5%)
B	29 (46.8%)
C	24 (38.7%)
NRS	
2	42 (67.8%)
3	18 (29%)
4	2 (3.2)
Total protein (g/L)	
Median ± SD	6.72 ± 0.52
Albumin (g/L)	
Median ± SD	3.33 ± 0.24
Prealbumin(g/dL)	
Median ± SD	0.20 ± 0.08
Transferrin(g/L)	
Median ± SD	2.50 ± 0.60

Nutritional status of the studied patients was estimated using Subjective Global Assessment (SGA) scale prior to hospitalization (during physical examination). The SGA evaluation included: weight history, food intake, gastrointestinal symptoms and changes in functional capacity as well as physical examination. The above-mentioned features were classified as follows: normal (0 score), mild (1+), moderate (2+) or severe (3+). Moreover, all patients were evaluated with the use of NRS (Nutritional Risk Score, NRS 2002), which takes into account the deterioration of nutritional status, the severity of the disease and patient age. The other studied parameters related to the assessment of the nutritional status were as follows: BMI (Body Mass Index) and laboratory test results (total serum protein, albumin, transferrin and prealbumin levels) [BMI, total protein and albumin were tested before the commencement of therapy (I) and after the termination of therapy (VII)]. Summarizing patients’ score and the results of both laboratory tests and physical examination, the patients’ nutritional status was defined. We divided patients into three groups: well-nourished (A), moderately malnourished (B) and severely malnourished (C) according to SGA scale. For the purpose of statistical analysis, we compared the SGA-A vs SGA-B and C as well as SGA-A and B vs SGA-C groups. Moreover, patients assigned to B or C groups were considered as a pre-cachectic or cachectic, respectively. All patients were consulted by a medical professional, who reviewed the SGA score with the patient to obtain answers to all questions regarding nutritional status (PG-SGA; Patient-Generated Subjective Global Assessment) and also completed physical examination to assess muscle wasting, loss of body fat and the presence of ankle and sacral edema and ascites.

The study was approved by the Bioethical Commission of the Medical University of Lublin (KE-0254/232/2014). All patients signed an informed consent prior to the study.

### Genotyping and ELISA

DNA was isolated from whole blood samples using DNA Blood Mini Kit (Qiagen, Canada). Genotyping was conducted using real-time PCR method and TaqMan SNP genotyping assay with allele discriminating software. The TaqMan fluorescently labeled probes (Applied Biosystems, USA) targeting the studied *TNF-α* SNP and Genotyping Master Mix (ThermoFisherScientific, USA) were used for DNA amplification in the StepOnePlus Real-Time PCR System (Applied Biosystems, USA). All genotyping steps were conducted under the conditions of protocol provided by the manufacturer.

Plasma TNF-α level was measured using TNF alpha Human ELISA Kit Ultrasensitive (Thermo Fisher Scientific, USA). The detection range was 0.2–32 pg/mL and the sensitivity was equal to the minimal detectable dose of this kit (< 0.09 pg/mL).

### Statistical analysis

Statistical analysis was conducted using MedCalc software version 12.7 (MedCalc Software, Belgium). The Fisher’s exact test and Chi-squared test were used to compare the distribution of clinical–demographic and nutritional factors among patients with different genotypes of *TNF-α*. Odds ratio (OR) with 95% Confidence Interval (95% CI) test was applied to assess risk of both genetic and clinical–demographic factors on nutritional status of patients. The differences in the analyzed factors among patients with different nutritional status and different *TNF-α* genotypes were analyzed by *U* Mann–Whitney rank sum test and ANOVA Kruskal–Wallis test. One-way analysis of variance (one-way ANOVA) was used to test the difference between the means of several subgroups of a variable. Prior to the ANOVA test, Levene’s test for equality of variances was performed. If the ANOVA test was positive (*p* < 0.05); then, a post hoc test (Student–Newman–Keuls’ test) was conducted for pairwise comparison of subgroups. Kaplan–Meier estimator and Cox-regression model were applied to assess factors [with hazard ratio calculation (HR)] affecting patients’ survival. The results with over median score were considered as high, whereas these below median range were assessed as low. Results with *p* value of less than 0.05 were considered as statistically significant.

## Results

The following distribution of *TNF-α* −1031T/C was achieved in the study group: CC in 6 patients (9.7% of the study group), CT in 19 patients (30.6%) and TT in 37 patients (59.7%), respectively. Distribution of −1031T/C genotype was within the Hardy–Weinberg equilibrium (*p* = 0.150). The median concentration of plasma TNF-α in the whole studied group was 9.62 ± 1.59 pg/mL.

First, we assessed the factors affecting the risk of malnutrition or cachexia according to SGA scale. We found that patients with performance status (PS) score greater than or equal to 1 point according to ECOG–WHO scale who simultaneously demonstrated weight loss of at least 5% of total body mass (BM) or carried CC genotype had higher risk of being assessed as cachectic compared to other cases (*p* = 0.019; OR = 3.724 and *p* = 0.044; OR = 9.737, respectively). C allele carriers (CC or CT genotype) also had over 13-fold higher risk to be assigned to SGA-C group compared to TT homozygous subjects (*p* = 0.0001). The factors affecting the risk of either malnutrition or cachexia are presented in Table 2. Subsequently, we divided patients into two groups regarding the use of parenteral nutrition intervention [parenterally nourished patients (PN) and patients without parenteral nutrition (WPN)], and then, we compared the distribution of nutritional and genetic factors between the studied cases. SGA-C patients were more often parenterally treated compared with SGA-A and/or SGA-B patients (*p* = 0.045). During the course of RTH, the PN patients increased their BM and BMI compared to WPN cases (*p* = 0.015 and *p* = 0.030, respectively) and also had significantly higher total plasma protein (TP) concentration (*p* = 0.043). As regards the examined factors, PN subjects had significantly higher TNF-α plasma concentration (*p* = 0.015) and more frequently carried CC genotype (4 patients; *p* = 0.015) (Supplementary file 1).

**Table 2:** Impact of the clinical–demographic, nutritional and genetic factors on the SGA scoring

Factor	A	B and C	*p* OR [95% CI]	A and B	C	*p* OR [95% CI]
SGA						
Gender						
Male	7 (13.7%)	44 (86.3%)	0.705 0.716 [0.127–4.03]	32 (62.6%)	19 (37.4%)	0.614 1.404 [0.377–5.231]
Female	2 (18.2%)	9 (81.8%)		6 (54.5%)	5 (45.5%)	
Age (years)						
≥ 63	7 (21.9%)	25 (78.1%)	0.107 3.92 [0.744–20.65]	20 (62.5%)	12 (37.5%)	0.840 1.111 [0.40–3.09]
< 63	2 (6.7%)	28 (93.3%)		18 (60%)	12 (40%)	
Performance status (PS)						
< 1	9 (16.4%)	46 (83.6%)	0.456 3.065 [0.161–58.35]	36 (65.5%)	19 (34.5%)	0.08 4.737 [0.839–26.76]
> 1	0	7		2 (28.6%)	5 (71.4%)	
Histopathological diagnosis						
Squamous-cell carcinoma	8 (14%)	49 (86%)	0.718 0.653 [0.064–6.614]	33 (57.9%)	24 (42.1%)	0.165 0.124 [0.007–2.355]
Others	1 (20%)	4 (80%)		5	0	
Disease stage						
I and III	2 (14.3%)	12 (85.7%)	0.978 0.976 [0.179–5.333]	10 (71.4%)	4 (28.6%)	0.380 1.786 [0.490–6.512]
IV	7 (14.6%)	41 (85.4%)		28 (58.3%)	20 (41.7%)	
Tumor location						
Upper throat	3 (17.6%)	14 (82.4%)	0.668 1.393 [0.306–6.333]	13 (76.5%)	4 (23.5%)	0.139 2.600 [0.733–9.217]
Lower throat	6 (13.3%)	39 (86.7%)		25 (55.6%)	20 (44.4%)	
Larynx	5 (14.7%)	29 (85.3%)	0.963 1.035 [0.250–4.287]	19 (55.9%)	15 (44.1%)	0.337 0.600 [0.212–1.702]
Others	4 (14.3%)	24 (85.7%)		19 (67.9%)	9 (32.1%)	
Alcohol consumption						
Yes	4 (14.3%)	24 (85.7%)	0.963 0.967 [0.233–4.001]	14 (50%)	14 (50%)	0.101 0.417 [0.147–1.185]
No	5 (14.7%)	29 (85.3%)		24 (70.6%)	10 (29.4%)	
Smoking status						
Smoker	8 (15.4%)	44 (84.6%)	0.661 1.636 [0.182–14.75]	31 (59.6%)	21 (40.4%)	0.539 0.633 [0.147–2.729]
Non-smoker	1 (10%)	9 (90%)		7 (70%)	3 (30%)	
Concurrent CTH						
Yes	2 (8.3%)	22 (91.7%)	0.284 0.403 [0.076–2.125]	13 (54.3%)	11 (45.7%)	0.362 0.615 [0.216–1.749]
No	7 (18.4%)	31 (81.6%)		25 (65.8%)	13 (34.2%)	
BMI (I) All patients						
< 24.9	4 (10%)	37 (90%)	0.148 0.346 [0.082–1.460]	21 (51.2%)	20 (48.8%)	0.028 4.048 [2.160–14.12]
> 25	5 (23.8%)	16 (76.2%)		17 (70.8%)	4 (29.2%)	
< 18.5	1 (10%)	9 (90%)	0.661 0.611 [0.068–5.510]	4 (40%)	6 (60%)	0.141 0.353 [0.088–1.414]
> 18.5	8 (15.4%)	44 (84.6%)		34 (65.4%)	18 (34.6%)	
Weight loss (I vs VII) All patients						
< 5%	2 (6.7%)	28 (93.3%)	0.107 0.255 [0.048–1.344]	23 (76.7%)	7 (23.3%)	0.019 3.724 [1.246–11.13]
> 5%	7 (21.9%)	25 (78.1%)		15 (46.9%)	17 (53.1%)	
< 10%	4 (10%)	36 (90%)	0.137 0.333 [0.078–1.416]	22 (55%)	18 (45%)	0.175 0.458 [0.149–1.414]
> 10%	5 (25%)	15 (75%)		16 (72.7%)	6 (27.3%)	
Genotype distribution of *TNF-α* −1031T/C						
CC	0	6	0.527 0.385 [0.020–7.420]	1 (11.1%)	5 (88.9%)	0.044 9.737 [1.061–89.40]
CT and TT	9 (16.1%)	47 (83.9%)		37 (66.1%)	19 (33.9%)	
TT	9 (24.3%)	28 (75.7%)	0.055 17.0 [0.941–306.99]	31 (83.8%)	6 (16.2%)	0.0001 13.29 [3.862–45.70]
CT and CC	0	25		7 (28%)	18 (72%)	

Secondly, we examined the distribution of *TNF-α* genotype according to both clinical–demographic and nutritional factors in the studied patients. We did not find any correlation between clinical–demographic features and the studied SNP (Supplementary file 2); however, we noted a correlation between the distribution of *TNF-α* SNP and the nutritional status of the studied patients. The subjects who carried CC genotype had significantly lower BM compared with both TT and CT genotype carriers (*p* = 0.045). A similar trend was observed in patients with the presence of C allele (CC or CT) compared to homozygous TT subjects (*p* = 0.044; median: 58 vs 64 kg, respectively). Moreover, homozygous CC subjects had the lowest plasma TP and albumin concentration among the studied patients (*p* = 0.036 and *p* = 0.048, respectively). Similar results were observed in patients with C allele positivity compared with TT carriers. Moreover, CC patients had the highest TNF-α plasma level (median: 10.70 ± 0.72 pg/mL, *p* = 0.006) among studied cases. CC and CT cases analyzed together had significantly higher TNF-α concentration compared to TT patients (*p* = 0.0015; median 9.98 vs 9.08 pg/mL, respectively). Genotype distribution of *TNF-α* −1031T/C according to patients’ nutritional factors is shown in Table 3. Similar results were obtained for comparison of means. However, this way of analysis was more powerful in discriminating CC genotype as unfavorable factor affecting the nutritional status of the studied patients (Table 4). We also examined the effect of studied polymorphism on the nutritional status of the studied group including separate analysis for PN and WPN patients (Table 5). Patients with CC genotype and patients with either CC or CT genotype (C allele carriers) were at a significantly higher risk of developing cachexia compared to other patients (*p* = 0.044; OR = 9.737 and *p* = 0.0001; OR = 13.29, respectively). This correlation with C allele positivity was also observed in PN and WPN patients (*p* = 0.003; OR = 7.714, *p* = 0.023; OR = 51.0, respectively). Moreover, homozygous CC were at an over 38-fold higher risk of scoring 3 or 4 points according to NRS compared to other genotype carriers (*p* = 0.015). C allele positivity also assigned both PN and WPN patients to higher NRS scoring. During the therapy, CC subjects had a significantly higher risk of BMI reduction (< 18.5) compared to CT and TT patients (*p* = 0.030; OR = 44.33 and *p* = 0.006; OR = 23.0).

**Table 3:** (Comparison of medians) *TNF-α* −1031T/C genotype distribution according to both clinical–demographic and nutritional factors of studied patients

Factor (median ± SD)	CC	CT	TT	*p*	CT and CC	TT	*p*
*TNF-α* −1031T/C							
Weight (kg) (I) all patients	55 ± 11.64	60 ± 7.07	64 ± 9.01	0.042	58 ± 7.83	64 ± 9.01	0.044
Weight (kg) (I) men	61 ± 8.21	63 ± 7.43	69 ± 10.99	0.049	61 ± 7.06	69 ± 10.99	0.012
Weight (kg) (I) women	53 ± 8.72	55 ± 9.52	62.5 ± 9.65	0.133	53.5 ± 8.24	62.5 ± 9.65	0.045
Weight (kg) (VII) all patients	51 ± 10.23	58 ± 8.67	60 ± 9.76	0.389	58 ± 9.02	60 ± 9.76	0.322
Weight (kg) (VII) men	60 ± 4.35	58 ± 8.85	60 ± 9.73	0.381	58.5 ± 8.22	60 ± 9.73	0.350
Weight (kg) (VII) women	51 ± 5.77	66 ± 8.49	55 ± 10.03	0.090	51 ± 11.64	55 ± 10.03	0.840
BMI (I) all patients	19.95 ± 4.49	22.83 ± 4.59	23.54 ± 4.07	0.122	22.71 ± 4.74	23.54 ± 4.07	0.179
BMI (I) men	23.09 ± 2.08	22.58 ± 4.19	24.09 ± 4.40	0.464	22.84 ± 3.97	24.09 ± 4.40	0.429
BMI (I) women	17.96 ± 3.73	20.85 ± 3.23	21.43 ± 3.73	0.186	19.95 ± 7.45	21.43 ± 3.73	0.093
BMI (VII) all patients	20.22 ± 4.95	19.53 ± 4.18	19.37 ± 3.67	0.866	19.53 ± 4.29	19.37 ± 3.67	0.908
BMI (VII) men	22.53 ± 3.05	19.53 ± 3.58	21.05 ± 3.81	0.343	20.07 ± 3.63	21.05 ± 3.81	0.860
BMI (VII) women	16.65 ± 2.91	18.81 ± 0.76	19.00 ± 3.76	0.348	18.52 ± 2.46	19.00 ± 3.76	0.315
Transferrin (g/L)	2.80 ± 0.47	2.50 ± 0.61	2.50 ± 0.60	0.279	2.60 ± 0.59	2.50 ± 0.60	0.421
Prealbumin (g/dL)	0.3 ± 0.05	0.2 ± 0.08	0.2 ± 0.08	0.355	0.25 ± 0.08	0.2 ± 0.08	0.178
TP (g/L) (I)	6.64 ± 0.53	6.73 ± 0.48	6.71 ± 0.55	0.985	6.73 ± 0.48	6.71 ± 0.55	0.943
TP (g/L) (VII)	5.83 ± 0.66	6.36 ± 0.59	6.46 ± 0.60	0.036	6.26 ± 0.56	6.46 ± 0.60	0.048
Albumin (g/L) (I)	3.19 ± 0.13	3.26 ± 0.20	3.38 ± 0.24	0.048	3.23 ± 0.19	3.38 ± 0.24	0.047
Albumin (g/L) (VII)	2.98 ± 0.62	3.11 ± 0.44	3.28 ± 0.38	0.031	3.04 ± 0.16	3.28 ± 0.38	0.013
TNF-α plasma level (pg/mL)	10.70 ± 0.72	9.76 ± 1.54	9.08 ± 1.49	0.006	9.98 ± 1.37	9.08 ± 1.49	0.0015

*I* Measurement conducted before the commencement of therapy, *VII* measurement conducted after the termination of therapy

**Table 4:** (Comparison of means) *TNF-α* −1031T/C genotype distribution according to both clinical–demographic and nutritional factors of studied patients

Factor (mean ± SD)	CC	CT	TT	*p*	CT and CC	TT	*p*
*TNF-α* −1031T/C							
Weight (kg) (I) all patients	54.65 ± 11.51	62.43 ± 11.29	66.22 ± 11.65	0.043	60.72 ± 11.32	66.22 ± 11.65	0.042
Weight (kg) (I) men	62.0 ± 10.0	63.76 ± 11.29	67.87 ± 11.65	0.238	63.65 ± 11.21	67.87 ± 11.65	0.198
Weight (kg) (I) women	49 ± 11.51	62.67 ± 10.69	75 ± 11.28	0.043	58.11 ± 11.03	75 ± 11.28	0.049
Weight (kg) (VII) all patients	55.67 ± 9.96	59.16 ± 9.54	60.54 ± 9.51	0.624	57.56 ± 9.54	60.54 ± 9.51	0.424
Weight (kg) (VII) men	60 ± 10	58.35 ± 11.29	61.20 ± 11.60	0.514	58.95 ± 9.54	61.20 ± 11.60	0.409
Weight (kg) (VII) women	47.67 ± 9.97	65.0 ± 9.35	57.17 ± 9.81	0.148	54.6 ± 9.42	57.17 ± 9.81	0.694
BMI (I) all patients	19.58 ± 4.42	23.28 ± 4.28	23.52 ± 4.45	0.125	22.40 ± 4.28	23.52 ± 4.45	0.336
BMI (I) men	22.28 ± 4.31	22.76 ± 4.28	24.08 ± 4.47	0.492	22.69 ± 4.28	24.08 ± 4.47	0.237
BMI (I) women	16.88 ± 4.42	22.85 ± 4.07	25.43 ± 4.33	0.043	19.28 ± 4.37	25.43 ± 4.33	0.250
BMI (VII) all patients	20.22 ± 3.96	20.66 ± 3.91	20.05 ± 3.91	0.851	20.55 ± 3.91	20.05 ± 3.91	0.609
BMI (VII) men	23.03 ± 4.31	20.79 ± 3.91	21.20 ± 3.91	0.369	21.29 ± 3.91	21.20 ± 3.91	0.920
BMI (VII) women	16.41 ± 3.96	19.84 ± 3.87	20.12 ± 3.96	0.134	17.78 ± 3.98	20.12 ± 3.96	0.100
Transferrin (g/L)	2.78 ± 0.40	2.58 ± 0.60	2.45 ± 0.59	0.255	2.58 ± 0.60	2.45 ± 0.59	0.400
Prealbumin (g/dL)	0.283 ± 0.09	0.253 ± 0.08	0.231 ± 0.08	0.262	0.261 ± 0.08	0.231 ± 0.08	0.945
TP (g/L) (I)	6.67 ± 0.51	6.68 ± 0.50	6.70 ± 0.52	0.985	6.68 ± 0.50	6.70 ± 0.52	0.866
TP (g/L) (VII)	5.69 ± 0.72	6.40 ± 0.66	6.53 ± 0.65	0.002	6.18 ± 0.66	6.53 ± 0.65	0.150
Albumin (g/L) (I)	3.19 ± 0.22	3.32 ± 0.24	3.46 ± 0.24	0.014	3.29 ± 0.24	3.46 ± 0.24	0.008
Albumin (g/L) (VII)	2.87 ± 0.43	3.09 ± 0.39	3.25 ± 0.40	0.044	3.06 ± 0.39	3.25 ± 0.40	0.040
TNF-α plasma level (pg/mL)	10.83 ± 1.34	9.67 ± 1.60	9.31 ± 1.57	0.002	10.71 ± 1.60	9.31 ± 1.57	< 0.001

*I* measurement conducted before the commencement of therapy, *VII* measurement conducted after the termination of therapy

**Table 5:** Impact of *TNF-α* −1031T/C gene polymorphism on the nutritional status of studied patients

Factor	CC	CT or TT	*p*, OR [95% CI]	TT	CT or CC	*p*, OR [95%CI]	CT	CC or TT	*p*, OR [95% CI]
SGA All patients									
A	0	9	0.527 0.385 [0.020–7.420]	9	0	0.055 17.0 [0.941–306.99]	0	9	0.108 0.093 [0.005–1.688]
B and C	6 (11.3%)	47 (88.7%)		28 (52.8%)	25 (47.2%)		19 (35.8%)	34 (64.2%)	
A and B	1 (2.6%)	37 (97.4%)	0.044 9.737 [1.061–89.40]	31 (81.6%)	7 (18.4%)	0.0001 13.29 [3.862–45.70]	6 (15.8%)	32 (84.2%)	0.0023 0.159 [0.048–0.519]
C	5 (20.8%)	19 (79.2%)		6 (25%)	18 (75%)		13 (54.2%)	11 (45.8%)	
SGA Without parenteral nutrition									
A	0	8	0.963 0.929 [0.041–21.16]	8	0	0.094 12.14 [0.657–224.57]	0	8	0.123 0.101 [0.005–1.865]
B and C	2 (4.9%)	39 (95.1%)		24 (58.5%)	17 (41.5%)		15 (36.6%)	26 (63.4%)	
A and B	1 (2.9%)	33 (97.1%)	0.554 0.424 [0.025–7.272]	27 (79.4%)	7 (20.6%)	0.003 7.714 [1.984–29.99]	6 (17.6%)	28 (82.4%)	0.005 0.143 [0.037–0.556]
C	1 (6.7%)	14 (93.3%)		5 (33.3%)	10 (66.7%)		9 (60%)	6 (40%)	
SGA Parenterally nourished									
A	0	1	0.790 0.630 [0.021–18.84]	1	0	0.317 5.667 [0.189–169.54]	0	1	0.790 0.630 [0.021–18.84]
B and C	4 (33.3%)	8 (66.7%)		4 (33.3%)	8 (66.7%)		4 (33.3%)	8 (66.7%)	
A and B	0	4	0.218 0.136 [0.006–3.254]	4	0	0.023 51.0 [1.705–1525.9]	0	4	0.218 0.136 [0.006–3.254]
C	4 (44.4%)	5 (55.6%)		1 (11.1%)	8 (88.9%)		4 (44.4%)	5 (55.6%)	
NRS All patients									
2 and 3	6 (10%)	54 (90%)	0.747 0.596 [0.026–13.83]	37 (61.7%)	23 (38.3%)	0.186 7.979 [0.367–173.59]	17 (28.3%)	43 (71.7%)	0.110 0.080 [0.04–1.762]
4	0	2		0	2		2	0	
2	0	42	0.015 38.10 [2.019–719.04]	31 (73.8%)	11 (26.2%)	0.002 6.576 [2.025–21.36]	11 (26.2%)	31 (73.8%)	0.273 0.532 [0.172–1.645]
3 and 4	6 (30%)	14 (70%)		6 (30%)	14 (70%)		8 (40%)	12 (60%)	
NRS Without parenteral nutrition									
2 and 3	2 (4.2%)	46 (95.8%)	0.299 0.161 [0.005–5.052]	32 (66.7%)	16 (33.3%)	0.285 5.909 [0.228–153.17]	14 (29.2%)	34 (70.8%)	0.237 0.140 [0.005–3.646]
4	0	1		0	1		1	0	
2	0	34	0.107 0.078 [0.004–1.739]	26 (76.5%)	8 (23.5%)	0.017 4.875 [1.326–17.92]	8 (23.5%)	26 (76.5%)	0.112 0.352 [0.097–1.274]
3 and 4	2 (13.3%)	13 (86.7%)		6 (40%)	9 (60%)		7 (46.7%)	8 (53.3%)	
NRS Parenterally nourished									
2 and 3	4 (33.3%)	8 (66.7%)	0.790 1.588 [0.053–47.52]	5 (41.7%)	7 (58.3%)	0.648 2.200 [0.075–64.91]	3 (25%)	9 (75%)	0.230 0.123 [0.004–3.782]
4	0	1		0	1		1	0	
2	0	8	0.023 0.020 [0.0-0.587]	5 (62.5%)	3 (37.5%)	0.08 17.29 [0.712–419.95]	3 (37.5%)	5 (62.5%)	0.512 2.40 [0.175–32.88]
3 and 4	4 (80%)	1 (20%)		0	5		1 (20%)	4 (80%)	
BMI (I) All patients									
< 24.9 (N and UW)	5 (12.2%)	36 (87.8%)	0.366 2.778 [0.303–25.46]	22 (53.7%)	19 (46.3%)	0.181 0.463 [0.150–1.431]	14 (34.1%)	27 (65.9%)	0.406 1.659 [0.503–5.475]
> 25.0 (OW)	1 (4.8%)	20 (95.2%)		15 (71.4%)	6 (28.6%)		5 (23.8%)	16 (76.2%)	
< 18.5 (UW)	3 (30%)	7 (70%)	0.033 7.0 [1.174–41.74]	4 (40%)	6 (60%)	0.176 0.384 [0.096–1.534]	3 (30%)	7 (70%)	0.962 0.964 [0.221–4.216]
> 18.5 (N and OW)	3 (5.8%)	49 (94.2%)		33 (63.5%)	19 (36.5%)		16 (30.8%)	36 (69.2%)	
BMI (I) Without parenteral nutrition									
< 24.9 (N and UW)	1 (3.1%)	31 (96.9%)	0.648 0.516 [0.030–8.805]	19 (59.4%)	13 (40.6%)	0.237 0.450 [0.120–1.691]	12 (37.5%)	20 (62.5%)	0.160 2.80 [0.665–11.79]
> 25.0 (OW)	1 (5.9%)	16 (94.1%)		13 (76.5%)	4 (23.5%)		3 (17.6%)	14 (82.4%)	
< 18.5 (UW)	0	7	0.962 2.80 [0.665–11.79]	4 (57.1%)	3 (42.9%)	0.626 0.667 [0.131–3.398]	3 (42.9%)	4 (57.1%)	0.453 1.875 [0.364–9.665]
> 18.5 (N and OW)	2 (4.8%)	40 (95.2%)		28 (66.7%)	14 (33.3%)		12 (28.6%)	30 (71.4%)	
BMI (I) Parenterally nourished									
< 24.9 (N and UW)	4 (44.4%)	5 (55.6%)	0.218 7.364 [0.307–176.42]	3 (33.3%)	6 (66.7%)	0.571 0.50 [0.045–5.514]	2 (22.2%)	7 (77.8%)	0.328 0.286 [0.023–3.524]
> 25.0 (OW)	0	4		2 (50%)	2 (50%)		2 (50%)	2 (50%)	
< 18.5 (UW)	3	0	0.030 44.33 [1.440-1365.15]	0	3	0.232 0.143 [0.006–3.471]	0	3	0.333 0.206 [0.008–5.051]
> 18.5 (N and OW)	1 (10%)	9 (90%)		5 (50%)	5 (50%)		4 (40%)	6 (60%)	
BMI (VII) All patients									
< 24.9 (N and UW)	6 (12.5%)	42 (87.5%)	0.320 4.435 [0.235–83.70]	28 (58.3%)	20 (41.7%)	0.690 0.778 [0.226–2.673]	14 (29.2%)	34 (70.8%)	0.641 0.741 [0.211–2.608]
> 25.0 (OW)	0	14		9 (64.3%)	5 (35.7%)		5 (35.7%)	9 (64.3%)	
< 18.5 (UW)	5 (33.3%)	10 (66.7%)	0.006 23.0 [2.416–218.95]	8 (53.3%)	7 (46.7%)	0.566 0.709 [0.220–2.291]	2 (13.3%)	13 (86.7%)	0.111 0.272 [0.055–1.349]
> 18.5 (N and OW)	1 (2.1%)	46 (97.9%)		29 (61.7%)	18 (38.3%)		17 (36.2%)	30 (63.8%)	
BMI (VII) Without parenteral nutrition									
< 24.9 (N and UW)	2 (5.1%)	37 (94.9%)	0.832 1.40 [0.062–31.47]	26 (66.7%)	13 (33.3%)	0.693 1.333 [0.319–5.570]	11 (28.2%)	28 (71.8%)	0.473 0.589 [0.139–2.499]
> 25.0 (OW)	0	10		6 (60%)	4 (40%)		4 (40%)	6 (60%)	
< 18.5 (UW)	2 (16.7%)	10 (83.3%)	0.07 17.86 [0.794–401.41]	8 (66.7%)	4 (33.3%)	0.909 1.083 [0.273–4.293]	2 (16.7%)	10 (83.3%)	0.240 0.370 [0.070–1.945]
> 18.5 (N and OW)	0	37		24 (64.9%)	13 (35.1%)		13 (35.1%)	24 (64.9%)	
BMI VII Parenterally nourished									
< 24.9 (N and UW)	4 (44.4%)	5 (55.6%)	0.218 7.364 [0.307–176.42]	2 (22.2%)	7 (77.8%)	0.094 0.095 [0.006–1.498]	3 (33.3%)	6 (66.7%)	0.765 1.50 [0.106–21.31]
> 25.0 (OW)	0	4		3 (75%)	1 (25%)		1 (35%)	3 (75%)	
< 18.5 (UW)	3	0	0.030 44.33 [1.440-1365.15]	0	3	0.232 0.143 [0.005–3.471]	0	3	0.333 0.206 [0.008–5.051]
> 18.5 (N and OW)	1 (10%)	9 (90%)		5 (50%)	5 (50%)		4 (40%)	6 (60%)	
Weight loss (I vs VII) All patients									
< 5%	4 (13.3%)	26 (86.7%)	0.356 2.308 [0.390–13.64]	18 (60%)	12 (40%)	0.960 1.026 [0.372–2.833]	8 (26.7%)	22 (73.3%)	0.511 0.694 [0.234–2.064]
> 5%	2 (6.2%)	30 (93.8%)		19 (59.4%)	13 (40.6%)		11 (34.4%)	21 (65.6%)	
< 10%	5 (12.5%)	35 (87.5%)	0.331 3.00 [0.328–27.46]	22 (55%)	18 (45%)	0.314 0.570 [0.191-1.70]	13 (32.5%)	27 (67.5%)	0.670 1.284 [0.407–4.047]
> 10%	1 (4.5%)	21 (95.5%)		15 (68.2%)	7 (31.8%)		6 (27.3%)	16 (72.7%)	
Weight loss (I vs VII) Without parenteral nutrition									
< 5%	2 (9.1%)	20 (90.9%)	0.227 6.707 [0.305–147.38]	14 (63.6%)	8 (36.5%)	0.825 0.875 [0.269–2.851]	6 (27.3%)	16 (72.7%)	0.648 0.750 [0.219–2.574]
> 5%	0	27		18 (66.7%)	9 (33.3%)		9 (33.3%)	18 (66.7%)	
< 10%	2 (6.5%)	29 (93.5%)	0.469 3.136 [0.143–69.02]	18 (58.1%)	13 (41.9%)	0.169 0.396 [0.106–1.482]	11 (35.5%)	20 (64.5%)	0.336 1.925 [0.508–7.298]
> 10%	0	18		14 (77.8%)	4 (22.2%)		4 (22.2%)	14 (77.8%)	
Weight loss (I vs VII) Parenterally nourished									
< 5%	2 (25%)	6 (75%)	0.571 0.50 [0.045–5.514]	4 (50%)	4 (50%)	0.295 4.00 [0.299–53.47]	2 (25%)	6 (75%)	0.571 0.50 [0.045–5.514]
> 5%	2 (40%)	3 (60%)		1 (20%)	4 (80%)		2 (40%)	3 (60%)	
< 10%	3 (33.3%)	6 (66.7%)	0.765 1.50 [0.106–21.31]	4 (44.4%)	5 (55.6%)	0.512 2.40 [0.175–32.88]	2 (22.2%)	7 (77.8%)	0.328 0.286 [0.023–3.524]
> 10%	1 (25%)	3 (75%)		1 (25%)	3 (75%)		2 (50%)	2 (50%)	

*I* Measurement conducted before the commencement of therapy, *VII* measurement conducted after the termination of therapy, *UW* underweight, *OW* overweight, *N* normal

**Fig. 1:**
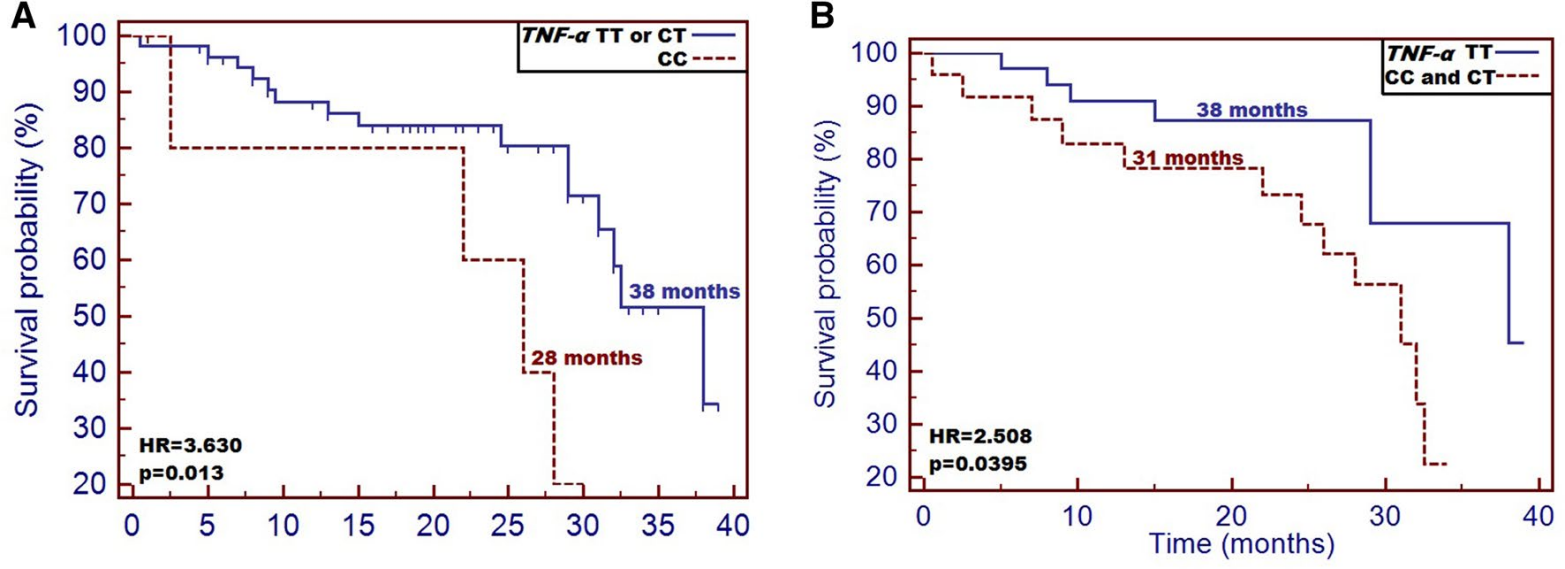
Impact of *TNF-α* −1031T/C SNP on patients’ overall survival: **a** differences in overall survival between groups of patients with CC and both TT and CT genotype; **b** differences in overall survival between groups of patients with C allele presence and TT homozygous patients

Finally, we examined the impact of both nutritional and studied factors on patients’ survival. Patients carrying CC genotype had significantly higher risk of early death and they also demonstrated significantly shorter overall survival (OS) [28 vs 38 months (HR = 3.630 [0.612–21.55]), *p* = 0.013)] compared to other genotype carriers (Fig. 1a). Analyzing OS for C allele carriers, similar results were observed compared to homozygous TT subjects. CC and CT patients analyzed together had significantly shorter OS compared to TT patients (median OS: 31 vs 38 months; HR = 2.508 [1.004–6.267], *p* = 0.0395) (Fig. 1b). Factors affecting patients’ survival are summarized in Table 6. Cox-regression model including all the patients’ data (demographic, clinical, nutritional and genetic factors) discriminated PS and CC genotype of *TNF-α* as most significant factors affecting lower OS in the study group (overall model fit *p* = 0.011) (Table 6).

**Table 6:** Factors affecting the overall survival of HNC patients in log-rank test and multivariate Cox logistic regression

Factor	Log-rank test	
	HR [95% CI]	*p*
Gender	1.171 [0.368–3.723]	0.777
Age	1.132 [0.452–2.837]	0.792
Smoking history	1.715 [0.515–5.712]	0.460
Alcohol consumption	1.301 [0.531–3.226]	0.550
Performance status	3.361 [0.60-18.831]	0.019
Tumor stage	1.881 [0.676–5.239]	0.303
Parenteral nutrition	1.617 [0.548–4.772]	0.322
SGA	2.818 [0.996–7.972]	0.126
NRS	1.890 [0.644–5.543]	0.271
BMI loss > 5% (I vs VII)	1.752 [0.695–4.415]	0.270
BMI loss > 10% (I vs VII)	1.436 [0.578–3.520]	0.443
Weight loss > 5% (I vs VII)	1.217 [0.489–3.032]	0.673
Weight loss > 10% (I vs VII)	1.374 [0.556–3.396]	0.492
Total protein (I)	1.227 [0.499–3.020]	0.653
Albumin (I)	1.726 [0.698–4.267]	0.228
TNF-α −1031T/C	4.960 [0.808–30.48]	0.019
Plasma TNF-α	2.582 [1.031–6.468]	0.028
Cox proportional-hazard regression model		
Performance status	3.10 [0.930–10.31]	0.047
*TNF-α* −1031T/C	2.142 [1.124–4.08]	0.021
Overall model fit *p* = 0.011, stepwise method		

*I* Measurement conducted before the commencement of therapy, *VII* measurement conducted after the termination of therapy

## Discussion

Malnutrition and cachexia are common among HNC patients and contribute to reduction of the quality of patients’ life, poorer therapy outcomes and higher risk of early death. Despite the recent advances in nutritional management, the molecular background of cancer cachexia is still disputable. The investigation of genetic factors, such as SNPs within genes encoding the proteins that regulate the inflammatory response still seems to be an attractive option for the selection of patients with high risk of malnutrition.

TNF-α is a pro-cachectic factor participating in the recruitment of inflammatory cells that subsequently considerably contribute to the degradation of muscle tissue proteins. TNF-α also increases gluconeogenesis and promotes the loss of adipose tissue (lipolysis). As a result of the above-mentioned processes, the inhibition of lipid, protein and glycogen synthesis is observed (Patel and Patel 2017; Tisdale 2002). Therefore, it is desirable to estimate what effects the molecular alterations have on TNF-α protein expression, because it could allow us to predict the grade of inflammatory response in cancer patients. Until today, several polymorphic variants of *TNF-α* have been described in literature as unfavorable factors responsible for intensification of cancer related inflammatory response. Marsha et al. investigated the following SNPs of *TNF-α*: -238 G/A and -308 G/A as malnutrition risk factors in patients with end-stage renal disease. *TNF-α* -308 AA and -238 AA genotype variants conferred 3.6-fold and 3.3-fold higher susceptibility and higher TNF-α levels in studied patients, respectively. Moreover, the presence of A allele positivity of -308G/A was associated with 2.3-fold higher risk of malnutrition compared to homozygous GG subjects. As regards -238 AA genotype, it was associated with 2.5-fold higher risk of death (Sarma et al. 2013). In another study concerning HNC patients, the AA haplotype of -308 G/A was associated with worse prognosis of the disease, shorter overall survival time and increased aggressiveness of the disease. According to the authors, the unfavorable cancer prognosis can be related to TNF-α protein level (Corrêaa et al. 2011). Recent large study performed by Johns et al. examined over 100 SNPs within genes related to cancer cachexia, and these molecular alterations were associated with both weight loss and muscle wasting in about 1200 studied individuals. Basing on a study set, the new cachexia related SNPs were revealed, and among them the *TNF-α* −1031T/C (Johns et al. 2017). The contribution of that SNP to the risk of cancer cachexia is still unknown; however, the results of the latest studies conducted in patients with various diseases demonstrated significant role of the discussed SNP in the mediation of systemic inflammatory response. Hernandez et al. conducted a systemic review followed by meta-analysis concerning *TNF-α* polymorphisms as the inflammatory markers of cardiovascular heart disease. The authors selected −1031T/C SNP as a risk factor of cardiovascular incidents (Hernández-Díaz et al. 2015). In the present study, we found that haplotype CC or the presence of C allele positivity are unfavorable factors associated with high risk of cachexia (9.70-fold and 13.29-fold higher risk) and poorer disease prognosis compared to TT genotype carriers. Moreover, patients carrying C allele demonstrated poorer nutritional status in terms of body mass, BMI, the result of laboratory tests and SGA scoring. We assumed that alterations of patients’ nutritional status were correlated with TNF-α level intensifying the inflammatory response in the studied individuals. In fact, patients carrying the CC genotype had significantly higher plasma TNF-α concentration compared to both CT and TT genotype carriers (10.70 pg/mL vs 9.76 pg/mL and 9.08 pg/mL, respectively) (*p* < 0.042). Most recently, Nourian et al. studied the −1031T/C SNP in patients with inflammatory bowel diseases (IBD). Interestingly, the authors found that CC haplotype was associated with genetic risk of IBD, and, moreover, mRNA expression of *TNF-α* was significantly higher in CC genotype carrying group compared to either CT or TT genotype carriers (Nourian et al. 2017). In the study of Negoro et al. high frequency of CC genotype of −1031T/C SNP was noted in patients with Crohn’s disease compared to individuals suffering from ulcerative colitis and healthy controls (Negoro et al. 1999). Similarly, Sanchez et al. demonstrated high prevalence of homozygous CC in the juvenile Crohn’s disease patients (Sanchez et al. 2009). High prevalence of CC genotype in patients with inflammatory diseases is probably the result of the increased TNF-α plasma level, which was demonstrated in our study. Moreover, we found that the occurrence of either CC genotype or C allele positivity are both unfavorable prognostic factors in HNC patients. Patients with CC genotype had significantly higher risk of early death compared to CT and TT haplotypes [28 vs 38 months (HR = 3.630 [0.612–21.55], *p* = 0.013)]. Our results are in accordance with Thiago et al. findings concerning AA of *TNF-α* −308G/A (Corrêaa et al. 2011). Perhaps, similarly to other promoter SNPs of *TNF-α*, −1031T/C is a potential regulator of TNF-α protein expression. Regarding the other promoter polymorphisms of *TNF-α*, the T allele of −863A/T has been linked to fat tissue accumulation and reduction of TNF-α serum level followed by decreased BMI and low body mass. On the other hand, the presence of A allele positivity of −308G/A was associated with increased protein production in various inflammatory diseases (Sharma et al. 2006; Hoffstedt et al. 2000; Tan et al. 2011). In our study set, the CC genotype carriers of −1031T/C had the highest TNF-α plasma concentration followed by the reduction of body weight and significantly decreased BMI (< 18.5) as well as poorer laboratory test results (the lowest plasma TP and albumin concentration) compared to either CT or TT genotype carriers. One of the limitations of our study was the use of a subjective tool (SGA scale) to nutritional status and occurrence of cachexia assessment.

We found that the presence of C allele positivity, and, especially, carrying the CC haplotype are both related to high risk of cancer cachexia in HNC patients. We also found, that the studied SNP significantly correlated with the plasma level of TNF-α and truly reflected the patients’ nutritional status. Moreover, *TNF-α* −1031T/C demonstrated its usefulness as a prognostic factor. Interestingly, despite the noted differences between SGA and NRS scoring, the occurrence of CC genotype was significant for both. It suggests high reliability of the studied SNP in objective assessment of patients’ nutritional status. Moreover, independently from the nutritional intervention with the use of parenteral nutrition, the PN patients and WPM patients who carried CC haplotype had significantly higher risk of cachexia in contrast to other genotype carriers (HR = 3.630 [0.612–21.55], *p* = 0.013). Probably, in the near future, patients burdened with CC genotype could be scheduled for pharmaceutical intervention with parenteral nutrition earlier; hence, they could be prevented from the development of severe malnutrition or cachexia. We are aware that our study was conducted on a small group of patients; therefore, the *TNF-α* −1031T/C should be further investigated in a larger study set to confirm its predictive and prognostic usefulness.
